# Correlation of *in vivo* and *ex vivo* ^1^H-MRI with histology in two severities of mouse spinal cord injury

**DOI:** 10.3389/fnana.2015.00024

**Published:** 2015-03-05

**Authors:** Harun N. Noristani, Nicolas Lonjon, Maïda Cardoso, Marine Le Corre, Emilie Chan-Seng, Guillaume Captier, Alain Privat, Christophe Coillot, Christophe Goze-Bac, Florence E. Perrin

**Affiliations:** ^1^Institute for Neurosciences of Montpellier, INSERM U1051Montpellier, France; ^2^Centre Hospitalier Régional Universitaire Montpellier, Gui de Chauliac HospitalMontpellier, France; ^3^Centre Hospitalier Régional Universitaire Montpellier, Lapeyronie Hospital, Chirurgie Orthopédique et Plastique PédiatriqueMontpellier, France; ^4^Charles Coulomb Laboratory (L2C-BioNanoNMRI team), UMR 5221 Centre National de la Recherche Scientifique -UniversityMontpellier, France; ^5^Department “Biologie-Mécanismes du Vivant,” Faculty of Science, University of MontpellierMontpellier, France

**Keywords:** spinal cord injuries, mice, *in vivo* MRI, *ex vivo* MRI, histology

## Abstract

Spinal cord injury (SCI) is a debilitating neuropathology with no effective treatment. Magnetic resonance imaging (MRI) technology is the only method used to assess the impact of an injury on the structure and function of the human spinal cord. Moreover, in pre-clinical SCI research, MRI is a non-invasive method with great translational potential since it provides relevant longitudinal assessment of anatomical and structural alterations induced by an injury. It is only recently that MRI techniques have been effectively used for the follow-up of SCI in rodents. However, the vast majority of these studies have been carried out on rats and when conducted in mice, the contusion injury model was predominantly chosen. Due to the remarkable potential of transgenic mice for studying the pathophysiology of SCI, we examined the use of both *in* and *ex vivo* ^1^H-MRI (9.4 T) in two severities of the mouse SCI (hemisection and over-hemisection) and documented their correlation with histological assessments. We demonstrated that a clear distinction between the two injury severities is possible using *in* and *ex vivo* ^1^H-MRI and that *ex vivo* MR images closely correlate with histology. Moreover, tissue modifications at a remote location from the lesion epicenter were identified by conventional *ex vivo* MRI analysis. Therefore, *in vivo* MRI has the potential to accurately identify in mice the progression of tissue alterations induced by SCI and is successfully implemented by *ex vivo* MRI examination. This combination of *in* and *ex vivo* MRI follow-up associated with histopathological assessment provides a valuable approach for further studies intended to evaluate therapeutic strategies on SCI.

## Introduction

Spinal cord injuries (SCI) are devastating neuropathologies that affect over 2.5 million patients worldwide, yield major handicaps and represent high costs to our society (from $1,031,394 to $4,373,912 per patient, National SCI Statistical Care). Depending on the spinal level and lesion severity, neurological impairments range from minimal sensory motor deficits to complete tetraplegia. Currently, there is no effective treatment for any symptoms associated with SCI.

To understand the pathophysiology of SCI and to explore the effectiveness of various therapeutic strategies, several animal models have been developed (Lee and Lee, 2013) accompanied with a large number of techniques (molecular biology, histopathology, functional analysis…) to evaluate the impact of neurological damage. Although both rats and mice have been used as preclinical models of SCI, the availability of transgenic mice makes them particularly valuable for studying the underlying pathophysiological mechanisms involved in SCI. Currently, accurate evaluation of the SCI extent in a living mouse remains a challenge. The most commonly used approach is histological examination; however, it makes the dynamic follow-up of SCI recovery on the same animal impossible.

Magnetic resonance imaging (MRI), a non-invasive method, provides the only means to assess the impact of an injury on the structure and function of the human spinal cord (Stroman et al., 2014; Wheeler-Kingshott et al., 2014). MRI is indeed well-established as the most commonly used imaging approach to diagnose and follow-up spinal cord injured patients (Do-Dai et al., 2010). Interestingly, *in vivo* MRI abnormalities have been correlated for almost 30 years with neurologic impairments (Kulkarni et al., 1988). Although *in vivo* MRI analysis was carried out in animal models of SCI as early as 1986 (Hackney et al., 1986), it is only during the last decade that MRI techniques have been effectively used to follow-up SCI in rodents (for reviews see Harel and Strittmatter, 2008; Denic et al., 2011) with however a strong predominance of studies in rats as compared to mice due to the field strength limitations. Indeed, the small size of the mice exemplify the challenges inherent to spinal cord imaging (Stroman et al., 2014) i.e., the small physical dimension of the spinal cord cross section, the physiological motion of the spinal cord in the spinal canal due to cerebrospinal fluid flow and the motion of the cord resulting from respiration and heartbeat. The first pioneer *in vivo* MRI follow-up in mice drew a comparison between MRI and histopathological examination (Wamil et al., 1998). Bonny et al. (2004) characterized for the first time parameters for time diffusion-weighted *in vivo* and *ex vivo* MRI in un-injured and SCI mice (Bonny et al., 2004). A further study in an ischemic model of mouse SCI evidenced changes in the dorsal horn that subsequently spreads to the ventral horn (Gaviria et al., 2006). *In vivo* (Bilgen et al., 2005) and *ex vivo* (Harrison et al., 2013) MRI analysis had also been reported in normal mouse spinal cord. SCI-related MRI studies were either associated with histological examination (Levene et al., 2008; Tatar et al., 2009), behavioral analysis (Kim et al., 2010) or both histological and behavioral analysis (Bilgen et al., 2007). Two studies using mouse contusion injury of the spinal cord analyzed concomitantly *in* and *ex vivo* MRI, histology and behavioral parameters (Nishi et al., 2007; Brennan et al., 2013), the first one allowed correlating lesion volumes with the force of impact on the spinal cord, spared white matter and functional recovery. The second one reported on the use of ultra-high field (16.4 T) *in vivo* DTI to quantitatively monitor the progression of white matter damage and linked DTI parameters to disruption of tissue integrity (Brennan et al., 2013).

Almost all *in vivo* MRI follow-ups in SCI mice were done using contusion injury models, whilst in the clinic, SCI patients display injuries with a wide range of severities. To closely mimic clinical settings it is mandatory to optimize *in vivo* MRI follow-up in several mouse models of SCI. *Ex vivo* MRI, on the other hand, allows the obtaining of images with high spatial resolution that, in turn, leads to more accurate assessment of tissue alterations. Thus, MRI technology has great translational potential by providing relevant anatomical and structural details non-invasively.

The aim of our study was (a) to follow-up lesion extension using *in vivo* MRI in two lesion severities of the mouse spinal cord (hemisection and over-hemisection), (b) to deepen our analysis, at the end of the experiment, of altered tissues using high resolution *ex vivo* MRI and (c) to evaluate putative correlation of *in* and *ex vivo* MRI assessments with histology in both injury severities.

## Materials and methods

### Animal and surgery

Adult Swiss males (3 months of age) were used (Charles Rivers, L'Arbresle, France). Animals were housed in controlled conditions with a standard environment of 12 h light/dark cycle in thermo regulated boxes and fed *ad libitum* with free access to drinking water. We carried out all animal experiments in accordance with the guidelines approved by the French Ministry of Agriculture and following the European Council directive (2010/63/UE). Every effort was made to minimize the number and suffering of animals.

Spinal cord injury (SCI) was done at thoracic level 9 (T9). Briefly, mice were anesthetised with an induction at 2% isoflurane and maintained throughout the surgery at 0.5% isoflurane (Aerane, Baxter, Deerfield, IL, USA). The skin and muscles overlying the low thoracic segment were cut along the back midline. T9 vertebra was removed. Two injury severities were done; “mid-severity” that consisted of a lateral hemisection (50%) and “high-severity” group where lesion was over hemisection with at least 80% of the total diameter of the cord injured. In both cases, the meninges were incised and the spinal cords were sectioned using a micro-scalpel (FST, Heidelberg, Germany). T9 level was chosen to obtain different levels of paraplegia (partial to complete depending on injury severity) whilst preserving full respiratory function. Five mice were used for the un-injured and mid-severity groups, while 3 were assigned to the high-severity group. We chose these two injury severities to recapitulate clinical settings, which display injuries of the spinal cord with a wide range of severities. Muscles and skin were then sutured and the animals were left to recover. Postoperative care: Bladder was emptied manually twice daily until the end of the study (high-severity group) or until they regained bladder control (mid-severity group).

### *In vivo* ^1^H-MRI

MRI experiments were done using a 9.4 Tesla apparatus (Agilent Varian 9.4/160/ASR, Santa Clara, California, USA) equipped with a MAGNEX TS1276D, a Quadrature Volume Coils 400 MHz RF43 (Rapid Biomedical, Rimpar, Germany) and associated with a VnmrJ Imaging acquisition system (Agilent, Palo Alto, California, USA). Anesthetic and animal holder system was used (MInerve Siemens A.G., Erlangen, Germany)/ RS2D (Haguenau, France). Mice were anesthetised using 2% isoflurane and monitored (respiration and temperature) using the MR-compatible Small Animal Monitoring and Gating System (Model 1025, SA Instruments, Inc., New York, USA). Respiration was maintained around 40 breaths/min by adjusting isoflurane level and oxygen flow rate. Longitudinal analysis was done at the following time points: 24 and 72 h, 1, 2, 3, 4, 5, and 6 weeks after lesion.

Axial images were obtained with a mems (Multiple Echo Multi Slices) protocol using the following parameters: TR = 1200 ms; TE = 10 ms; NE = 2; AVG4; FOV = 30 × 30 mm; 28 slices; thickness: 0.6 mm; gap = 0 mm; acquisition matrix (N_READ_ × N_PHASE_) = 256 × 256.

As acquisition was synchronized with respiration (controlled through levels of isoflurane and oxygen) to reduce motion artifacts, repetition time depended on breathe period (T_BREATHE_), typically about 2 s. Consequently, the scanning time was approximately 35 min (T_BREATHE_ × N_PHASE_ × AVG). Sagittal images were also obtained with a mems protocol using the following parameters: TR = 1200 ms; TE = 10 ms; NE = 2; AVG2; FOV = 40 × 30 mm; 10 slices, thickness: 0.6 mm; gap = 0 mm; acquisition matrix = 256 × 256. As previously, due to respiration gating, scanning time was approximately 15 min. All MRI visualization and segmentation were done using Myrian Sofware (Intrasense, Montpellier, France).

### Tissue processing

Six weeks after lesion, both injured and un-injured control mice were deeply anaesthetized with intraperitoneal injection of tribromoethanol (500 mg/kg), they received a transcardial perfusion of cold 0.1M phosphate buffer saline (PBS) at pH 7.2 followed by 4% paraformaldehyde (Sigma, UK) in PBS. Entire spinal cords were then dissected and post-fixed in the same fixative for 2 h.

To enhance contrast for *ex vivo* MRI acquisition, spinal cords were then incubated in gadolinium (1:100 in 0.1 M PBS; Dotarem, 0.5 mmol/ml, Guerbet, Roissy CdG, France) for 48 h. Just prior to imaging, spinal cords were placed in a 5-mm-diameter glass tube filled with Fluorinert FC-77 liquid (3M™ Electronic Liquids, Saint Paul, USA) that is a proton-free fluid with low water solubility and similar magnetic susceptibility to the tissue. This greatly reduces the background noise and artifacts from the surrounding medium during image acquisition. At the end of the *ex vivo* MRI acquisition, spinal cords were first rinsed in 0.1 M PBS, cryoprotected in sucrose 30%, included in Tissue Teck (Sakura, Alphen aan den Rijn, The Netherlands), frozen and kept at -80°C until processing.

### *Ex vivo* ^1^H-MRI

Spinal cords in a 5-mm-diameter glass tube filled with Fluorinert were placed in the 9.4 Tesla apparatus and imaged. Axial images were done with a mems protocol using the following parameters: TR = 1155 ms; TE = 14 ms; NE = 1; AVG = 180; FOV = 10 × 10 mm; 60 slices, thickness: 0.6 mm; gap = 0 mm; acquisition matrix = 256 × 256. The average was determined to satisfy Rose criterion (CSNR>5) (Rose, 1948). Scanning time: approximately 15 h.

### Histology

For histological analysis, frozen spinal cords were cut (14 μm) and mounted onto Superfrost Plus© slides (Thermo Fisher Scientific, Illkirch, France). Histological analysis was performed on serial sections throughout the whole spinal cord segments that underwent *ex vivo* MRI.

To observe the overall structure integrity of the spinal cord and to estimate lesion extension, we used two series of sections stained with Luxol Fast Blue and Toluidine Blue, respectively. For Luxol Fast Blue staining, slides were placed in 95% ethanol for 5 min followed by incubation in 0.1% Luxol Fast Blue for 12 h at room temperature. Sections were then rinsed in milli Q water (1 min), placed in 0.05% lithium carbonate (1 min) and washed in tap water (1 min). Slides were then placed in 0.5% neutral red solution (10 min), 100% ethanol (6 min) and two washes of xylene (5 min each). For Toluidine Blue staining, slides were placed in 0.1% Toluidine Blue solution (3 min) followed by 10 seconds in 100% ethanol and two washes of xylene (3 min each). All slides were then cover-slipped using Entellan (Merck KGaA, Germany) and were left to dry overnight.

To investigate the reaction of glial cells (astrocytes and microglia), we used immunohistochemistry with antibody against glial fibrillary acidic protein (GFAP, a classical astrocyte marker) and ionized calcium-binding adapter molecule 1 (Iba1, a macrophage/microglia-specific marker). For immunostaining, slides were first incubated for 20 min in 20 mM lysine followed by 1 h incubation in 1% bovine serum albumin solution (BSA, Sigma) and 0.1% Triton (Triton X-100, Sigma) in 0.1 M PBS. Slides were then incubated in either rabbit polyclonal IgG directed against GFAP (1:1000, Dako, Glostrup, Denmark) or rabbit anti Iba1 (1:1000, Wako Pure Chemical Industries, Osaka, Japan). The primary antibody was diluted in 0.1 M PBS supplemented with 1% BSA and 0.1% Triton. Following incubation for 24 h at room temperature, the slides were rinsed with 0.1 M PBS for 30 min and incubated in 1:1000 dilutions of biotinylated donkey anti-rabbit IgG (Jackson Immunoresearch, Stratech Scientific Ltd, Soham, UK) for 1 h at room temperature. Slides were rinsed with 0.1 M PBS followed by incubation for 30 min in avidin-biotin peroxidase complex (Vector Laboratories Ltd, Peterborough, UK). The peroxidase reaction product was visualized by incubating in a solution containing 0.022% of 3,3′diaminobenzidine (DAB, Aldrich, Gilligham, UK) and 0.003% hydrogen peroxide (H_2_O_2_) for 30 min. The reaction was stopped by rinsing the sections in 0.1 M PBS for 15 min. Slides were then dehydrated in ascending concentration of ethanol (50, 70, 80, 90, 95, and 100%) and finally xylene. Cover slips were applied using Entellan (Merck KGaA, Germany) and slides were left to dry overnight. To ensure the specificity of the staining, negative controls were used in which the primary antibody was not applied (data not shown).

Morphometric bright field photographs were obtained and analyzed using NanoZoomer RS slide scanner (NanoZoomer Digital Pathology System and NDP view software, Hamamatsu, Japan). The lesion area was measured as % of the total surface area (μm^2^) in a given spinal cord section using NDP view software. Similarly, the area of un-damaged tissue within the white and gray matters had been measured to estimate lesion extension in two injury severities.

### Statistics

Four different statistical tests had been chosen (Supplementary Table 1). For overall longitudinal *in vivo* MRI analysis of lesion volume in the 3 groups (un-injured, mid- and high severities) Two-Way analysis of variance (ANOVA) had been applied, for evolution of lesion volume over time per severity using *in vivo* MRI, One-Way ANOVA had been chosen, for comparison of several modalities of quantification (*in vivo, ex vivo*, and histology) on the same animal group paired t test was adapted whereas for comparison between groups unpaired t test was. Statistical tests were used according to the data analyzed. Statistical significance: ^*^P < 0.05, ^**^P < 0.01, ^***^P < 0.001. Means are presented with standard error of the means (SEM).

## Results

### Longitudinal *in vivo* ^1^H-MRI follow-up discriminates between mid- and high-severity of spinal cord injury

**Figure 1:**
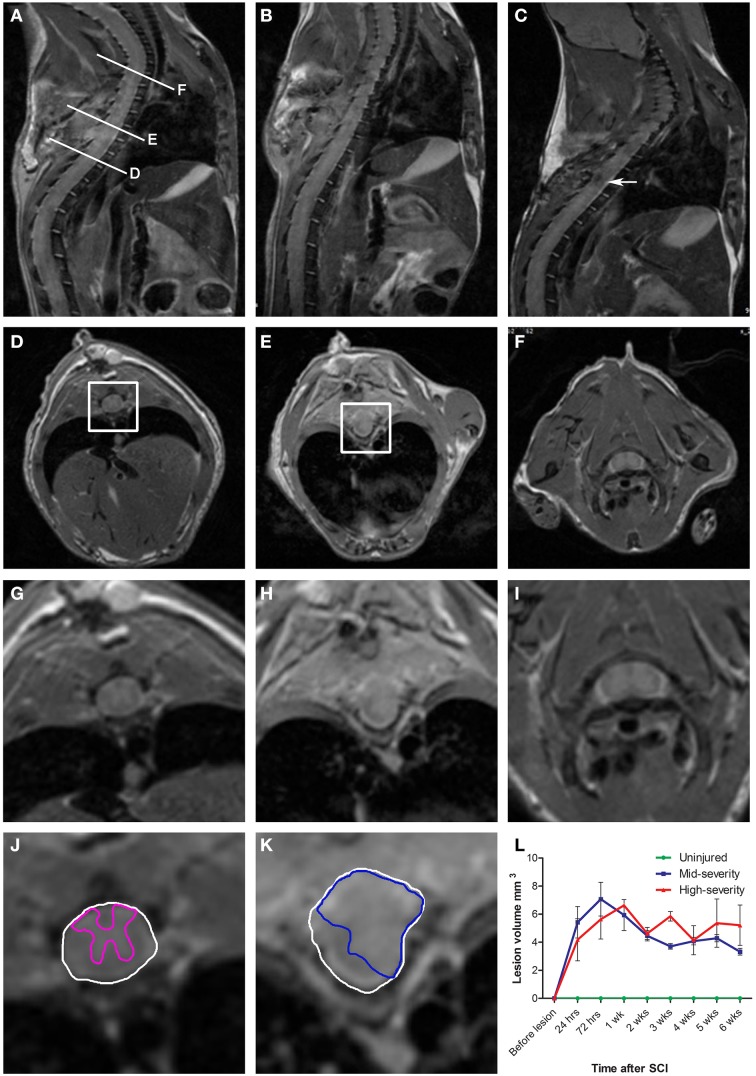
**Longitudinal*in vivo*^1^H-MRI assessments of two severities of spinal cord injury in mice. (A–C)** *In vivo* sagittal images of the same mouse that underwent a mid-severity lesion. **(A)** 24 h following spinal cord injury, **(B)** 1 week and **(C)** 6 weeks after SCI. **(D–F)** *In vivo* axial images taken at levels **(D,E,F)** indicated in **(A)**. **(G-I)** Zoom of *D*-*F*. **(D,G,J)** Below the lesion. **(E,H,K)** Lesion epicenter. **(F,I)-** Above the lesion. **(J,K)** Correspond to boxes in panels **(D,E)**. Drawings of the spared gray matter (**J**, *pink*) in the entire spinal cord (**J,K**, *white*) and of the injured tissue (**K**, *blue*). **(L)** Longitudinal quantification of the lesion volume in the two severities of SCI.

**Figure 2:**
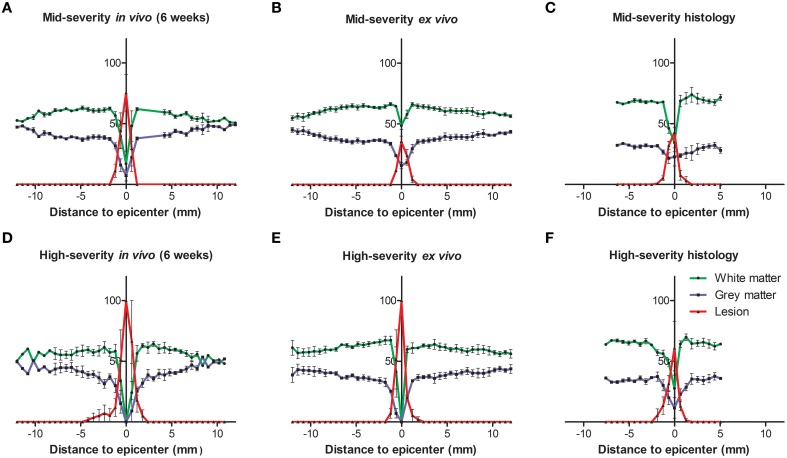
**Injured tissues quantification 6 weeks following both severities of SCI evaluated by histology and *in* and *ex vivo*^1^H-MRI**. In all panels the white matter is represented in green, the gray matter in blue and the lesion in red. **(A–C)** Mid-severity. **(D–F)** High severity. **(A,D)** *In vivo* MRI, **(B,E)** *ex vivo* MRI and **(C,F)** histological assessment 6 weeks after SCI.

**Figure 3:**
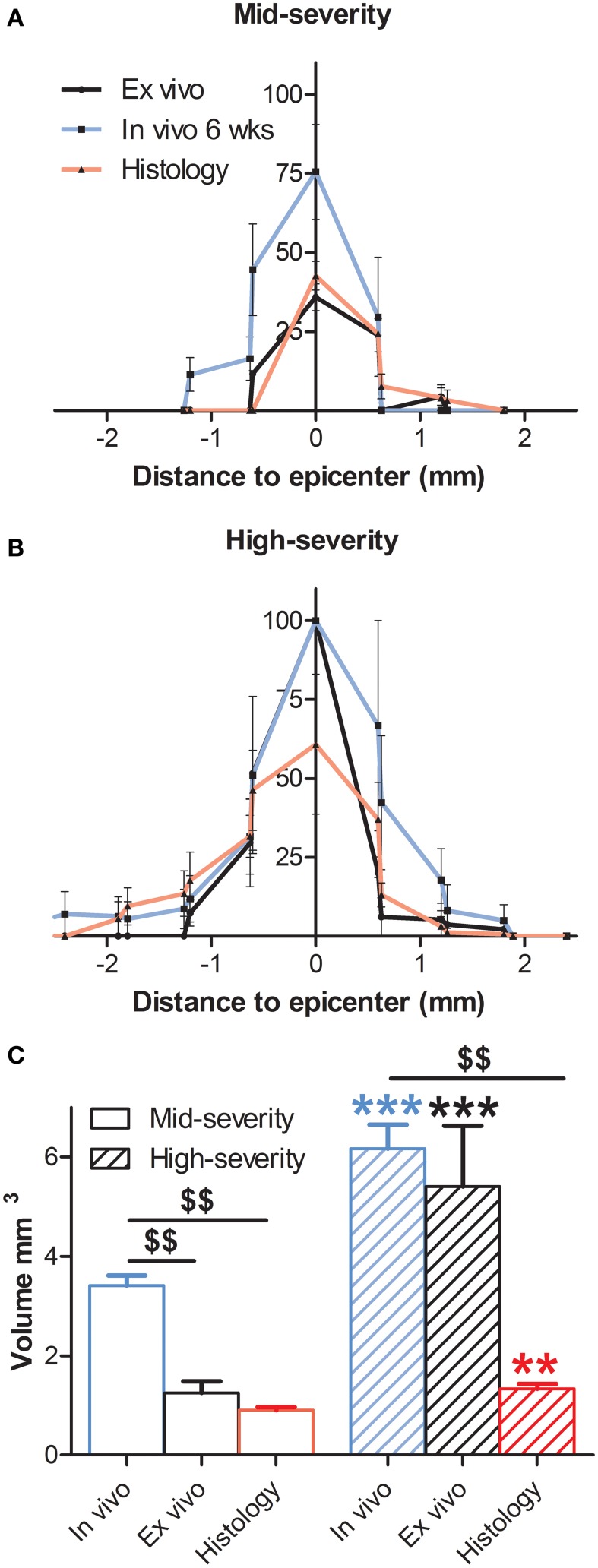
**Comparison of percentage of the lesion area at the epicenter and lesion volume 6 weeks following both severities of SCI evaluated by histology and *in* and *ex vivo*^1^H-MRI**. In all panels *in vivo* data are in blue, *ex vivo* data in black and histological data are represented in red. **(A)** Mid-severity. **(B)** High severity. **(C)** Lesion volume. ^$^ Comparison within the same severity group; ^*^ Comparison in between severity groups; ^**^ and ^$$^ *p* < 0.05; ^***^ *p* < 0.005.

Longitudinal follow-up of lesion extension using sagittal and axial *in vivo* ^1^H-MRI was obtained for each animal from 24 h to 6 weeks after injury for the two lesion severities (Figures 1A–L). We clearly identified modifications within the lesion site over time where at 24 h after lesion a hyper-intense peri-hematomal region appeared (Figures 1A,E) and persisted up to 1 week post-injury (Figure 1B). This most likely reflects a vasogenic oedema due to leakage of plasma resulting from blood-spinal barrier disruption and/or extracellular fluid accumulation such as blood or cerebrospinal fluid (CSF) leakage. At chronic stage, i.e., 6 weeks following SCI, the lesion site appeared with a hypo-intense region (Figure 1C, arrow). At the lesion epicenter, the spinal cord extends dorsally (Figures 1E,H,K) due to the absence of the posterior part of the vertebra resulting from surgical procedure. Since accurate evaluation of the lesion extension on sagittal views is not possible (Figures 1A–C), we acquired 28 axial images (0.6 mm thick) over a 1.6 cm segment centered on the lesion epicenter (Figures 1D–K). Lesion segmentation was carried out manually through volume quantification by outlining the spared and injured white and gray matters on axial images (Figures 1J–L, Table 1). Multiple comparison tests did not show overall differences between lesion severities (Figure 1L, Two-Ways ANOVA, Supplementary Table 1). However, analysis of lesion volume over time in each lesion severity revealed a peak at 72 h following injury in the mid-severity model whereas no significant difference was seen in the high-severity group (Figure 1L, One-Way ANOVA, Supplementary Table 1). To further compare with *ex vivo* ^1^H-MRI and histological lesion assessments, we carried out a more in-depth analysis of *in vivo* MRI data at 6 weeks following lesion (Figure 2). Mean percentages of damaged tissues at the epicenter were 75.5% ± 33.6 and 100% ± 0.0 in the mid- and high-severity groups, respectively (Figures 2A,D, 3A,B, Table 1). In the un-injured spinal cord at thoracic levels, the gray matter represents approximately 40% of the surface area of the entire spinal cord whereas the white matter accounts for the remaining 60% (Figure 2). Both gray and white matters were totally damaged following high-severity lesion whereas approximately 19% of the gray and 28% of the white matters remained intact in the mid-severity group, respectively (Figures 2A,D, 3A,B, Table 1). Along the rostro-caudal axis, lesion extension was 1.32 mm in mid-severity group and 3 mm in high-severity group (Figures 2A,D, 3A,B, Table 1). At 6 weeks after SCI, mid-severity groups showed a significantly smaller lesion volume compared to high-severity group (3.4 ± 0.2 mm^3^ and 6.2 ± 0.5 mm^3^, *p* = 0.0008, Figure 3C, Table 1). Thus, axial *in vivo* ^1^H-MRI allows precise discrimination in lesion evolution between mid- and high injury severities from 72 h post-surgery.

**Table 1:** **Comparison of lesion assessment using the three modalities (*in vivo* MRI, *ex vivo* MRI and histology) for both lesion severities**.

	**Mid-severity SCI**			**High-severity SCI**		
	***In vivo* MRI (6 weeks)**	***Ex vivo* MRI**	**Histology**	***In vivo* MRI (6 weeks)**	***Ex vivo* MRI**	**Histology**
% of the lesion at epicenter	75.5 ± 33.6	35.8 ± 9.5	42.6 ± 9.1	100 ± 0.0	100 ± 0.0	60.8 ± 38.5
% injured white matter at epicenter	71.6	19.2	42.4	100	100	53.9
% spared white matter at epicenter	28.4	80.8	57.6	0	0	46.1
% injured grey matter at epicenter	81.3	60.7	43.1	100	100	71.2
% spared grey matter at epicenter	18.7	39.3	56.9	0	0	28.8
Lesion extension (mm)	1.32 ± 0.3	1.44 ± 0.3	0.79 ± 0.3	3 ± 2.6	2 ± 0.9	2.5 ± 1.2
Lesion volume (mm^3^)	3.4 ± 0.2	1.2 ± 0.5	0.9 ± 0.1	6.2 ± 0.5	5.4 ± 2.1	1.3 ± 0.2

### *Ex vivo* ^1^H-MRI assessment discriminates between mid- and high-severity of spinal cord injury

**Figure 4:**
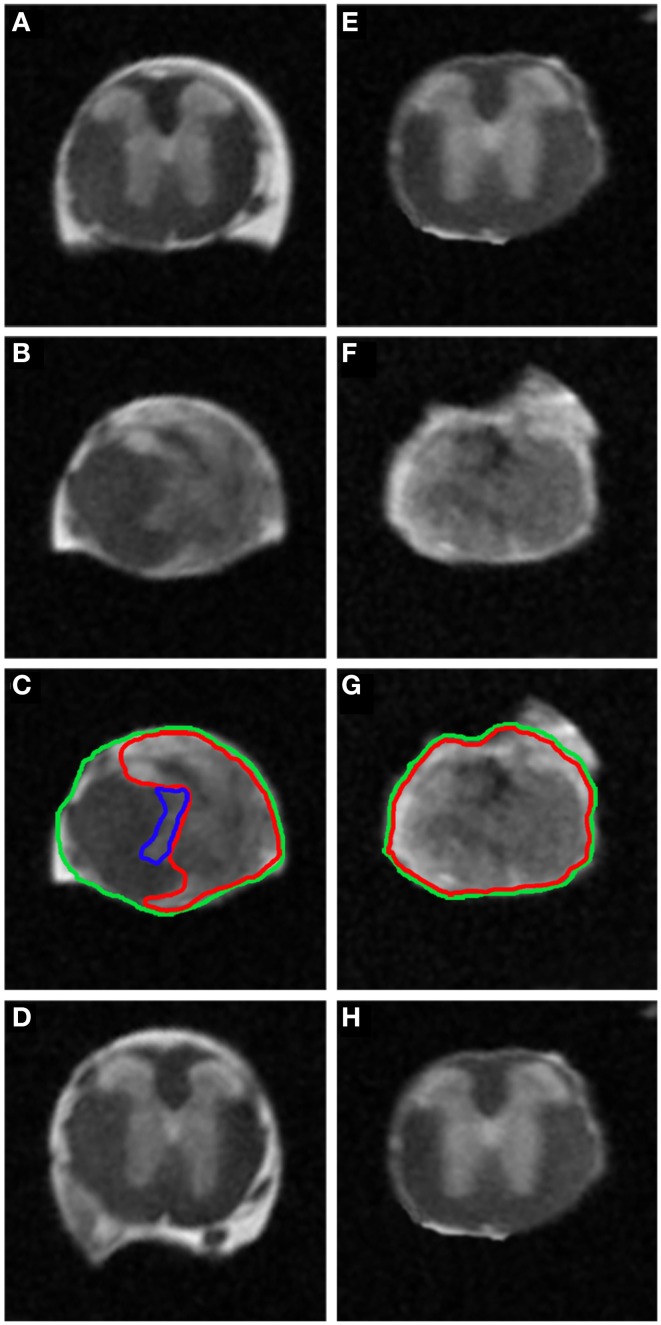
***Ex vivo*^1^H-MRI assessments 6 weeks following two severities of spinal cord injury**. MRI image of *ex vivo* mouse spinal cord after mid- **(A–D)** and high-injury severity **(E–H)**. Note the excellent anatomical resolution of the spinal cord in the lesion epicenter **(B,C,F,G)**, above **(A,E)** and below the lesion site **(D,H)**. Such high SNR and high isotropic resolution were obtained following incubation of the spinal cords in the Gadolinium and Fluorinert as well as long scanning time.

In an attempt to improve the discrimination between the spared and injured tissue after injury, we performed *ex vivo* ^1^H-MRI analysis that gives a higher resolution of magnetic resonance images. Improvement of image quality using *ex vivo* MRI as compared to *in vivo* MRI results from (a) an amplification of signal-to-noise ratio due to pre-incubation of the spinal cord in gadolinium that augments the contrast, (b) inclusion of Fluorinert during image acquisition that reduces background noise and (c) longer imaging time (15 h). Sixty axial images (0.6 mm thick) on a 3.6 cm segment centered on the lesion epicenter were taken. Assessment of lesion extension using *ex vivo* ^1^H-MRI at 6 weeks after surgery demonstrated clear differences between mid- (Figures 4A–D) and high-severity lesions (Figures 4E–H). A hyper-intense signal, corresponding to injured tissue covered half of the spinal area at the epicenter in mid-severity group (Figures 4B,C outlined in red) and the entire cord in high-severity group (Figures 4F,G outlined in red), respectively. Lesion volume quantification was carried out by outlining the spared and injured white and gray matters manually (Figures 4C,G) that showed a significant decrease in lesion volume in mid- compared to high-severity group (1.25 ± 0.2 mm^3^ and 5.4 ± 1.2 mm^3^, respectively, *p* = 0.0047, Figure 3C, Table 1, Supplementary Table 1). Further in-depth analysis of *ex vivo* ^1^H-MRI data revealed that the mean percentage of damaged tissues at the epicenter were of 35.8% ± 9.5 and 100% ± 0.0 in the mid- and high-severity groups, respectively (Figures 2B,E, 3A,B, Table 1). Both gray and white matters were totally damaged following high-severity lesion whereas in the mid- severity group 39% of the gray- and 80% of the white matters were spared (Figures 2B,E, 3A,B). Along the rostro-caudal axis, lesion extensions were 1.4 and 2 mm in the mid- and high-severity groups, respectively (Figures 2B,E, 3A,B, Table 1). Interestingly, we observed reduced signal intensity in the gray matter distal (both rostral and caudal) to the lesion epicenter when compared with un-injured animals (Supplementary Figure 1).

Therefore, axial *ex vivo* ^1^H-MRI analysis permitted us to clearly discriminate lesion extension between the mid- and high-severity of SCI with however some differences with the *in vivo* examination.

### Histological assessment discriminates between mid- and high-severity of spinal cord injury

**Figure 5:**
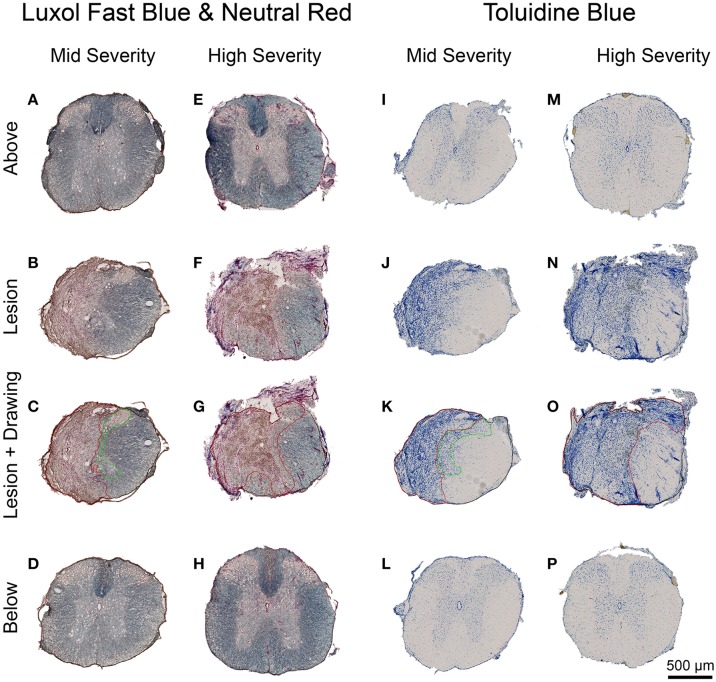
**Lesion extension histological assessment 6 weeks following two degrees of spinal cord injury**. Bright field micrographs displaying Luxol Fast Blue and Toluidine Blue staining after mid- **(A–D,I–L)** and high- **(E–H,M–P)** injury severities of the spinal cord. Scale bar **(A–P)**: 500 μm.

Luxol Fast Blue and Toluidine Blue staining were used to analyse the overall structural integrity of the spinal cord and to estimate lesion extension in the two injury severities. Luxol Fast Blue revealed myelin (in blue) in the white matter of the spinal cord above and below the injury site in the two injury severities (Figures 5A,D,E,H) as well as in spared white matter adjacent to the lesion site (Figures 5B,C,F,G). The lesion site was clearly identifiable at low magnification with a large number of neutral red-positive cells (Figures 5B,C,F,G). It displayed a small area of cavitation surrounded by disordered tissue in both injury severities (Figures 5B,C,F,G). As expected, the lesion area was increased in high- compared to mid-injury severity (Figures 5B,C,F,G). No differences in Luxol Fast Blue staining were observed between above and below the lesion sites in the two injury severities (Figures 5A–H).

In the intact tissue, Toluidine Blue staining predominantly labeled neurons in the gray matter of the spinal cord in the two groups (Figures 5I,L,M,P). By contrast, the lesion area displayed a high density of small Toluidine Blue-positive profiles in both gray and white matters (Figures 5J,K,N,O). Mid-injury severity group displayed a smaller lesion surface area compared to high-injury severity (Figures 5J,K,N,O). Toluidine Blue staining was similar between above and below the lesion sites in the mid- and high-injury severities (Figures 5I,L,M,P). Mean percentages of the damaged tissues at the lesion epicenter were 42.6% ± 9.1 and 60.8% ± 38.5 in the mid- and high-severity groups, respectively (Figures 5J,K,N,O, Table 1). Approximately 29% of the gray and 54% of the white maters were spared in the high-severity group (Figure 5, Table 1), whereas approximately 57% were spared in both gray and white matters in the mid-severity group. Along the rostro-caudal axis, lesion extensions were 0.8 and 2.5 mm in the mid- and high-severity groups, respectively (Figures 2C,F, 3A,B, Table 1).

High magnification examination of the sections revealed that immersion of the tissues in Gadolinium and Fluorinert (for *ex vivo* MRI) still allowed further classical histological observation of spinal cord tissues (Kouyoumdjian et al., 2009).

**Figure 6:**
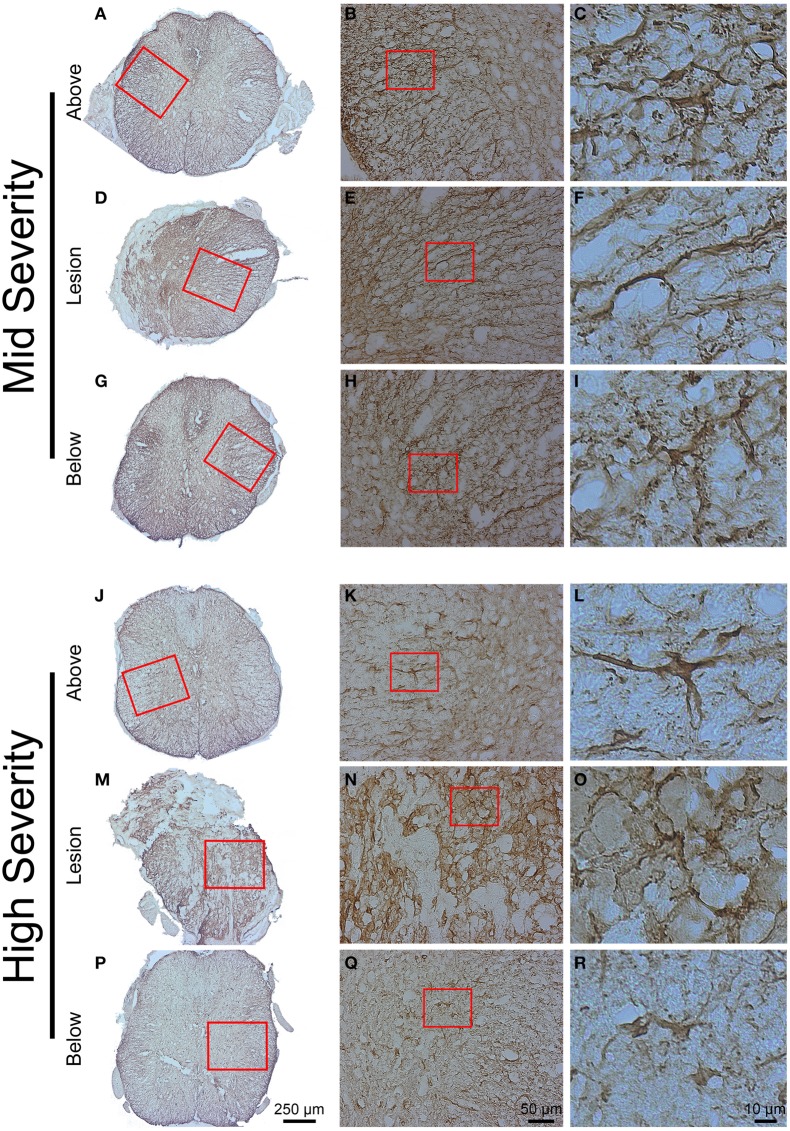
**Astrocytic reactivity 6 weeks following two degrees of spinal cord injury**. Bright field micrographs displaying GFAP-positive astrocytes after mid- **(A–I)** and high- **(J–R)** injury severities of the spinal cord. Astrocytes displayed typical stellate morphology with multiple non-overlapping branched processes both above and below the lesion sites **(C,I,L,R)**, whilst reactive astrocytes with elongated morphology and numerous over-lapping processes were localized adjacent to the lesion site **(F,O)**. Scale bar **(A,D,G,J,M,P)**: 250 μm, **(B,E,H,K,N,Q)**: 50 μm, **(C,F,I,L,O,R)**: 10 μm.

GFAP and Iba1 immunohistochemistry were used to analyse glial reactivity in the 2 injury severities. GFAP detection evidenced a heterogeneous distribution of astrocytes in the intact tissue with a higher density in the white matter compared to the gray matter both above and below the lesion site in mid- and high-injury severities (Figures 6A–C,G–I,J–L,P–R). In the mid-severity model, increased GFAP intensity was particularly evident in the spared tissue adjacent to the lesion site that isolated the damage site from surrounding tissue (Figures 6D–F). No GFAP labeling was obvious within the lesion center in both injury severities (Figures 6D–F,M–O). Morphologically, astrocytes displayed a stellate shape with multiple non-overlapping branched processes both above and below the lesion sites (Figures 6C,I,L,R). Reactive astrocytes localized adjacent to the lesion site mostly displayed an elongated morphology with close proximity to one another and numerous over-lapping processes (Figures 6F,O). Comparisons between the 2 injury severities did not display major differences in GFAP immunoreactivity and astrocyte morphology above and below the lesion sites.

**Figure 7:**
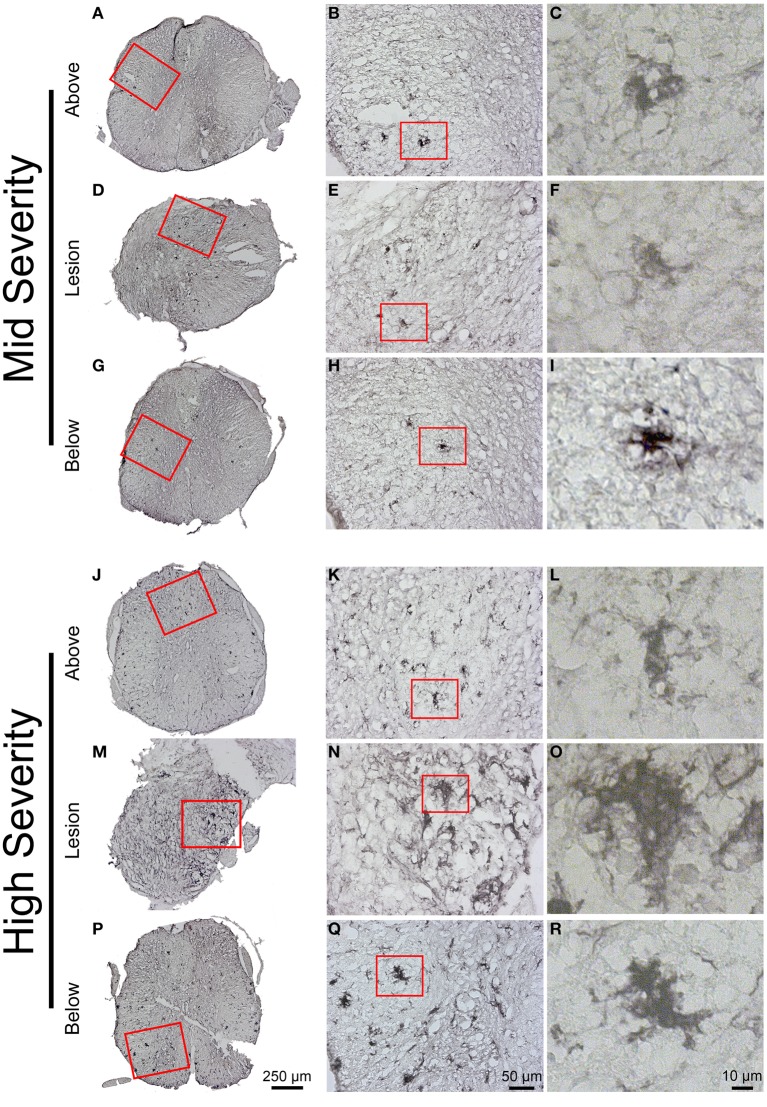
**Microglial reactivity 6 weeks following two degrees of spinal cord injury**. Bright field micrographs displaying Iba1-positive microglia after mid- **(A–I)** and high- **(J–R)** injury severities of the spinal cord. Microglia displayed typical ramified morphology with short and thin processes both above and below the lesion sites **(K,L)**, whilst activated/amoeboid microglia with enlarged cell bodies and thick processes were localized adjacent to the lesion site **(F,O)**. Scale bar **(A,D,G,J,M,P)**: 250 μm, **(B,E,H,K,N,Q)**: 50 μm, **(C,F,I,L,O,R)**: 10 μm.

Immunohistochemical staining using Iba1 antibody demonstrated a heterogeneous microglial distribution in the intact tissue above and below the lesion with a higher density within the gray matter (Figure 7). Increased Iba1 immunoreactivity was particularly evident within the lesion center and areas immediately adjacent to the lesion site (Figures 7D,M), which was particularly apparent in high-severity injury (Figure 7M). Morphologically, Iba1-positive microglia could be categorized into two groups: (a) ramified or resting microglia with small cell bodies emitting thin-to-medium processes that were randomly distributed (Figures 7K,L), and (b) activated/amoeboid microglia that displayed enlarged cell bodies with short and thick processes (Figures 7B,C,E,F,H,I,N,O,Q,R). Activated/amoeboid microglia predominated at the lesion site (Figures 7E,N), whereas both activated and ramified microglia were found distally (Figures 7B,H,K,Q). Comparisons between the 2 severities displayed increased density of activated/amoeboid microglia not only within the lesion site (Figures 7E,N), but also distal to the injury in high- compared to mid-injury severity (Figures 7B,H,K,Q).

Taken together, these data suggest that whereas astrocytes show no major differences in GFAP immunoreactivity between the two injury severities microglial reactivity, as seen with Iba1 immunoreactivity, is increased in high- compared to mid-injury severity.

### Comparison between *in vivo* ^1^H-MRI, high resolution *ex vivo* ^1^H-MRI and histological observations

We then examined putative correlation, 6 weeks after SCI in both injury severities, between the 3 modalities of analysis i.e., *in vivo* MRI at the end of the experiment, *ex vivo* MRI and histological data. At the epicenter, the lesion area (1 single section corresponding to the epicenter) showed no statistical difference in each group in any of the comparisons between the three modalities (Table 1, Supplementary Table 1). The lesion area at the epicenter appeared smaller in the mid-severity compared to high-severity group but only when using *ex-vivo* MRI (*p* = 0.0108). To deepen our analysis we analyzed whether there was a preferential damage at the epicenter between the white and the gray matters of the spinal cord. The only significant difference that was seen in the mid-severity model when using *ex vivo* MRI, was that the white matter appeared less damaged than the gray matter (14.8 vs. 60.7%, Supplementary Table 1). As lesion volume is more representative of the actual tissue damage than the only epicenter, we first compared lesion volumes between the 2 injury severities using the 3 analysis modalities. Lesion volume was smaller in the mid- as compared to high-severity model for all modalities (Figure 3C, Table 1, Supplementary Table 1). Comparison between modalities for a given group revealed that in the mid-severity group, *in vivo* MRI volume was higher than when assessed by *ex vivo* MRI and histology, whereas *ex vivo* and histological measures were similar. In the high-severity group, only *in vivo* MRI volume was higher than when assessed by histology (Figure 3C, Table 1, Supplementary Table 1). Even if *ex vivo* volume appeared larger than histological volume, this did not reach statistical significance (Supplementary Table 1) most likely due to the low number of animals and a high heterogeneity in the *ex vivo* measurement for the high-severity group (Figure 3C). Taken together, these results show that at the lesion epicenter there is no significant difference between the two injury severity groups in the evaluation of damaged tissues using the 3 modalities, suggesting that analysis at the epicenter only is not discriminative. Lesion volume shows the best correlation with injury severity using the 3 analysis methods. Evaluation of lesion volume is higher using *in vivo* MRI than both *ex vivo* MRI and histology in the mid-severity paradigm. In the high-severity group lesion volume is also higher using *in vivo* MRI than histological assessment.

## Discussion

Challenges with *in vivo* MR images analysis of the injured mouse spinal cord rely on the accurate identification of the lesioned and spared tissues. Image quality is indeed hampered by the small dimension of the spinal cord associated with physical motions. Synchronization of the acquisition with respiratory cycle greatly improves image quality but substantially increases acquisition time (Stroman et al., 2014). This drawback is particularly true in SCI due to fragility of the injured animal to repeated anesthetic procedures and has to be taken into account when setting-up acquisition parameters. In our study, using longitudinal (8 time points) axial *in vivo* ^1^H-MRI acquisition, we were able to discriminate lesion volume evolution between two injury severities (hemisection and over-hemisection) from 72 h until 6 weeks post-lesion. At 24 h after surgery and up to 5 weeks following injury MR images depicted increased signal intensity at the lesion site and in its close vicinity that most likely reflects an oedematous or cystic lesion (Byrnes et al., 2010). In both injury severities, at 6 weeks after surgery, small focal hypo-intense signals were seen within the lesion area that may be indicative of fibrotic tissues, as already reported in contusion injury in mice (Bilgen et al., 2007). This signal modification may also be due to time course hemorrhage and in particular hemoglobin degradation (Cosnard et al., 2003). *Ex vivo* ^1^H-MRI provides a better image quality and more clearly discriminates between the mid- and high-severity SCI. Conversely to *in vivo* ^1^H-MRI, at the lesion epicenter, distinction between severities was possible based on two criteria: lesion area and preferential damage of the gray matter in the mid- severity group. This confirm that *ex vivo* ^1^H-MRI allows detection of subtle morphological changes resulting from pathological processes (Gaviria et al., 2006). We have shown that soaking tissues in Gadolinium followed by Fluorinert increases signal-to-noise-ratio without greatly impairing further classical histological examinations. Interestingly, we observed reduced T2 signal in the gray matter distal to the lesion site as compared to signal in spinal cords of un-injured animals. Reduced T2 signal intensity showed a good correlation with low cell density and increased GFAP immunoreactivity (Supplementary Figure 1). Our results suggest that conventional *ex vivo* MRI analysis should not only focus on the lesion area but it should also be extended to more distal segments of the spinal cord following injury. In two previous studies using *ex vivo* diffusion tensor imaging after SCI in rats, changes in diffusivity were reported extending 3 mm away from the lesion epicenter for up to 12 weeks after injury (Schwartz et al., 2003) and throughout the entire spinal cord for up to 25 weeks following contusion (Ellingson et al., 2008). Thus, even if conventional ^1^H-MRI conveys limited, if zero, information on axonal integrity following SCI (Schwartz and Hackney, 2003), *ex vivo* ^1^H-MRI can reveal altered tissue properties remote from the lesion epicenter. Comparisons between *ex vivo* MR images and histological analysis of the spinal cords revealed a good correlation. MRI closely matches the anatomical information provided by Toludine Blue staining and glial immunoreactivity including cystic cavities formation and distorted tissue at the lesion epicenter. Interestingly, microglia at variance with astrocytes show differential reactivity in the lesion severities 6 weeks after lesion. Finally, lesion volumes are similar when assessed by *ex vivo* and histology whereas volumes are higher in both lesion severities when evaluated by *in vivo* ^1^H-MRI. This certainly reflects partly a functional status of the tissues since *ex vivo* ^1^H-MRI and histology are done on fixed spinal cords.

## Conclusion

In this study, we have documented the correlation between *in* and *ex vivo* ^1^H-MRI and histology in two lesion severities of the mouse spinal cord. As reported for SCI in rats (Berens et al., 2005), *ex vivo* ^1^H-MR images and histologic assessments confirmed pathologic events observed *in vivo*. We show that (a) the 3 analysis modalities allow a clear distinction between the lesions, (b) *ex vivo* ^1^H-MRI analysis closely correlates with histology and, (c) that remote tissue modifications are readily identified by conventional *ex vivo* ^1^H-MRI. Therefore, combination of the 3 analysis modalities makes a very useful tool for the identification of tissues alteration after different severities of spinal cord section in mice. Such a combination is likely to permit a more predictive value to preclinical studies of therapeutic strategies of SCI in mouse models.

### Conflict of interest statement

The authors declare that the research was conducted in the absence of any commercial or financial relationships that could be construed as a potential conflict of interest.
