# Supplementary material for: Correlation of in vivo and ex vivo 1H-MRI with histology in two severities of mouse spinal cord injury
Source: Front Neuroanat. 2015 Mar 5;9:24. doi: 10.3389/fnana.2015.00024 (PMC4350395; doi:10.3389/fnana.2015.00024)
Supplement: Supplementary file 1 [file Table1.DOCX]

| **Type** | **Statistical test** | **Data compared** | **Results** | **Figure** |
| --- | --- | --- | --- | --- |
| Volume *in vivo* | 2 ways ANOVA | Uninjured / mid- severity | *** All time points except 6 wks ** | **1** |
| Volume *in vivo* | 2 ways ANOVA | Uninjured / high-severity | *** All time points | **1** |
| Volume *in vivo* mid-severity | 2 ways ANOVA | Mid / high-severity | NS | **1** |
| Volume *in vivo* high-severity | 1 way ANOVA | Mid-severity | * or ** From72 hrs. F = 7.343, R = 0.6851 | **1** |
| Volume *in vivo* | 1 way ANOVA | High-severity | NS | **1** |
| Mid-severity extension | 2 ways ANOVA | *Ex vivo* / *In vivo* 6 weeks | ** or *** at epicenter | **2&3** |
| Mid-severity extension | 2 ways ANOVA | *Ex vivo* / histology | NS | **2&3** |
| Mid-severity extension | 2 ways ANOVA | *In vivo* 6 weeks/ histology | ** or *** at epicenter | **2&3** |
| High-severity extension | 2 ways ANOVA | *Ex vivo* / *In vivo* 6 weeks | ** at 0.6 mm from epicenter | **2&3** |
| High-severity extension | 2 ways ANOVA | *Ex vivo* / histology | * at epicenter | **2&3** |
| High-severity extension | 2 ways ANOVA | *In vivo* 6 weeks / histology | * at epicenter | **2&3** |
| Mid-volume severity | Paired t test | *Ex vivo* / *In vivo* 6 weeks | ** | **3** |
| Mid-severity volume | Paired t test | *In vivo*/ histology | ** | **3** |
| Mid-severity volume | Paired t test | *Ex vivo* / histology | NS | **3** |
| High-severity volume | Paired t test | *Ex vivo* / *In vivo* 6 weeks | NS | **3** |
| High-severity volume | Paired t test | *In vivo* / histology | ** | **3** |
| High-severity volume | Paired t test | *Ex vivo* / histology | NS | **3** |
| *In vivo* volume severity | Unpaired t test | Mid / high-severity | *** | **3** |
| *Ex vivo* volume severity | Unpaired t test | Mid / high-severity | ** | **3** |
| Histology volume severity | Unpaired t test | Mid / high-severity | * | **3** |
| Mid-severity epicenter | Paired t test | *Ex vivo* / histology | NS | **3** |
| Mid-severity epicenter | Paired t test | *Ex vivo* / *In vivo* 6 weeks | NS | **3** |
| Mid-severity epicenter | Paired t test | *In vivo*/ histology | NS | **3** |
| High-severity epicenter | Paired t test | *Ex vivo* / histology | NS | **3** |
| High-severity epicenter | Paired t test | *Ex vivo* / *In vivo* 6 weeks | NS | **3** |
| High-severity epicenter | Paired t test | *In vivo* / histology | NS | **3** |
| *In vivo* epicenter severity | Unpaired t test | Mid / high-severity | NS | **3** |
| *Ex vivo* epicenter severity | Unpaired t test | Mid / high-severity | * | **3** |
| Histology epicenter severity | Unpaired t test | Mid / high-severity | NS | **3** |
| *In vivo* mid-severity WM vs GM | Paired t test | *In vivo* mid. WM/GM | NS | **2** |
| *In vivo* high-severity WM vs GM | Paired t test | *In vivo* high. WM/GM | NS | **2** |
| *Ex vivo* mid-severity WM vs GM | Paired t test | *Ex vivo mid*. WM/GM | ** | **2** |
| *Ex vivo* high-severity WM vs GM | Paired t test | *Ex vivo* high. WM/GM | NS | **2** |
| Histo mid-severity WM vs GM | Paired t test | Histo *mid*. WM/GM | NS | **2** |
| Histo high-severity WM vs GM | Paired t test | Histo high. WM/GM | NS | **2** |
| Mid-severity WM *ex vivo*/*in vivo* | Unpaired t test | WM mid severity *ex*/*in* | NS | **2** |
| Mid-severity GM *ex vivo/in vivo* | Unpaired t test | GM mid severity *ex*/*in* | NS | **2** |
| High-severity WM *ex vivo/in vivo* | Unpaired t test | WM high severity *ex*/*in* | NS | **2** |
| High-severity GM *ex vivo/in vivo* | Unpaired t test | GM high severity *ex*/*in* | NS | **2** |
| Mid-severity WM *ex vivo*/histo | Unpaired t test | WM mid severity *ex*/histo | NS | **2** |
| Mid-severity GM *ex vivo*/histo | Unpaired t test | GM mid severity *ex*/histo | NS | **2** |
| High-severity WM *ex vivo*/histo | Unpaired t test | WM high severity *ex*/histo | NS | **2** |
| High-severity GM *ex vivo*/histo | Unpaired t test | GM high severity *ex*/histo | NS | **2** |
| Mid-severity WM histo/*in vivo* | Unpaired t test | WM mid severity histo/*in* | NS | **2** |
| Mid-severity GM histo/*in vivo* | Unpaired t test | GM mid severity histo/*in* | NS | **2** |
| High-severity WM histo/*in vivo* | Unpaired t test | WM high severity histo/*in* | NS | **2** |
| High-severity GM histo/*in vivo* | Unpaired t test | GM high severity histo/*in* | NS | **2** |

**Supplementary Table 1:** Statistical tests used according to the data analyzed. *: P<0.05, **: P<0.01, ***: P<0.001. Means are presented with SEM. WM: white matter, GM: grey matter.
