# Supplementary figures and images for: Correlation of in vivo and ex vivo 1H-MRI with histology in two severities of mouse spinal cord injury
Source: Front Neuroanat. 2015 Mar 5;9:24. doi: 10.3389/fnana.2015.00024 (PMC4350395; doi:10.3389/fnana.2015.00024)

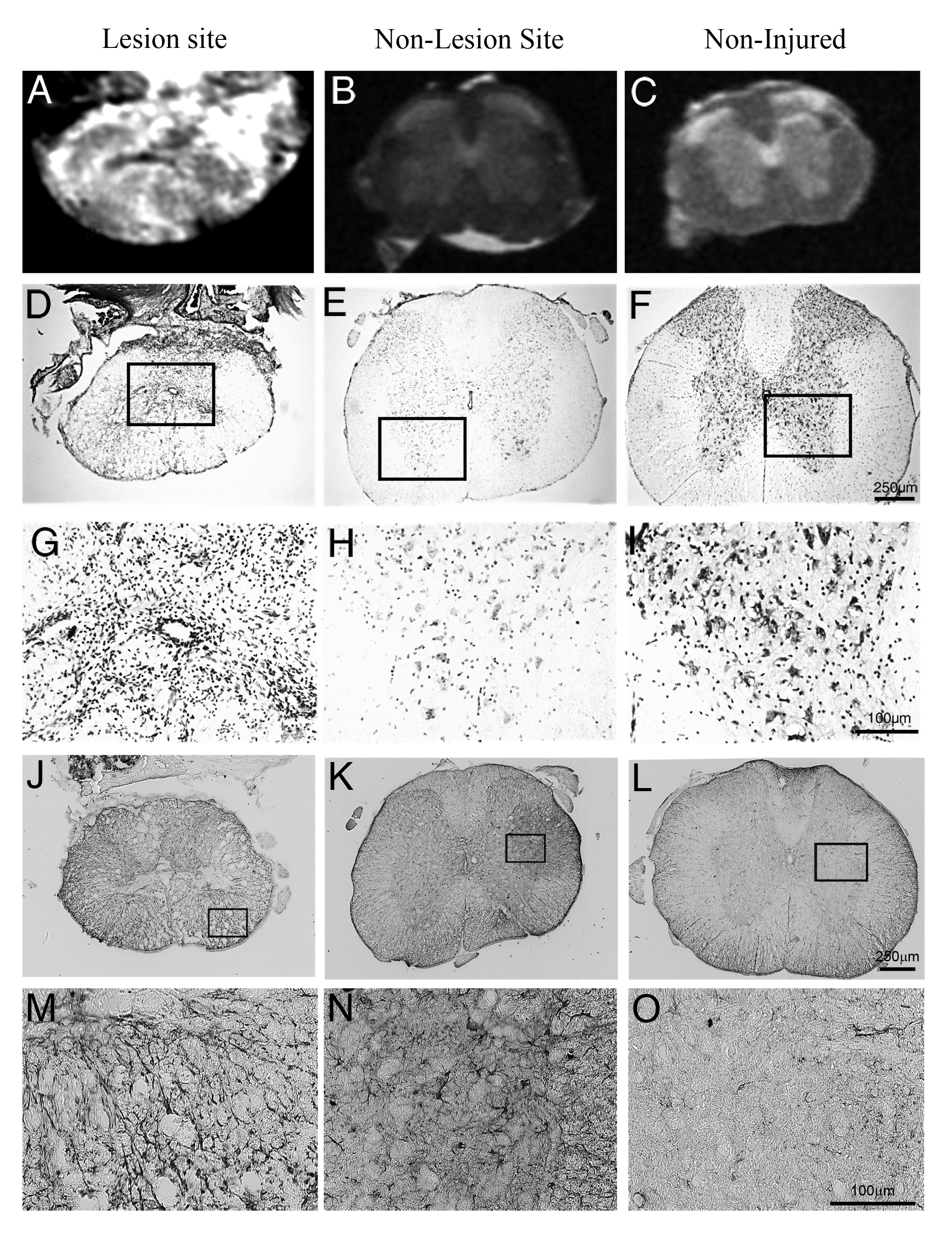

Supplement: Supplementary file 2 [file Image1.JPEG]
